# Development and Implementation of an Automated Electronic Maternal Early Warning System (E-MEWS) in a Level IV Obstetric Unit

**DOI:** 10.7759/cureus.84740

**Published:** 2025-05-24

**Authors:** Cesar R Padilla, Brendan Carvalho, Ann M Oakeson, Edward T Riley, Gillian Abir

**Affiliations:** 1 Anesthesiology, Perioperative and Pain Medicine, Stanford University School of Medicine, Stanford, USA; 2 Clinical Informatics, Lucile Packard Children’s Hospital Stanford, Palo Alto, USA

**Keywords:** early warning system, electronic health records, maternal mortality, monitoring, obstetric anesthesiology, obstetric labor complications

## Abstract

Maternal morbidity and mortality are increasing in the United States of America (USA), and automated early-warning surveillance systems have been suggested as a strategy to detect and reduce maternal morbidity and mortality. We report the development and implementation of a Maternal Early Warning System (MEWS) and automated alert notification platform into the electronic medical record (EMR) at the level IV obstetric unit at Lucille Packard Children's Hospital Stanford (Palo Alto, California, USA). This has been named E-MEWS. The development of the E-MEWS triggers and alert system occurred in three phases, each phase incorporating changes following analysis of interim audits. Here, we demonstrate the logistics involved in implementing MEWS into an EMR, including potential modifications required for operational functionality. The impact on maternal morbidity and mortality was not measured in this study.

## Introduction

Maternal mortality has increased 2.4-fold in the United States of America (USA) in the past 30 years, and the USA is ranked the highest amongst high-income countries in maternal mortality rates [1]. The majority of maternal deaths are attributed to indirect medical etiologies (e.g., cardiovascular disease), reflecting an increasingly complex patient population with a high burden of comorbidities [2]. Approximately 60% of maternal deaths are preventable, signaling the importance of developing screening tools as a strategy to identify patients at risk of clinical decompensation [3].

Maternal Early Warning Systems (MEWSs) were developed because standard adult warning tools are suboptimal for obstetric patients as they do not consider physiologic changes of pregnancy [4]. MEWSs aim to focus attention towards patients with vital sign abnormalities, directing clinicians towards patients most likely to benefit from clinical interventions before an adverse outcome occurs [4]. As a strategy to prevent maternal morbidity and mortality, the Society for Maternal-Fetal Medicine, for example, has called for the creation and development of electronic surveillance systems integrated with electronic medical records (EMRs) [5,6]. However, there is limited literature exploring the successful implementation of these recommendations [5].

In this study, we describe the implementation and optimization of an automated electronic alert notification platform integrated in the EMR using MEWS criteria (which we have named E-MEWS). The primary aim was to determine the expected number of alerts based on vital sign data captured in the EMR using MEWS triggers, while creating an operationally functional alert notification system during the intrapartum period. Our process did not seek to measure the effect of our automated system on patient outcomes.

## Materials and methods

This study was a quality improvement initiative conducted at the Lucile Packard Children’s Hospital Stanford (Palo Alto, California, USA), exempt from the Stanford University Institutional Review Board approval. 

In 2019, the Clinical Informatics Team (CIT) at Lucile Packard Children’s Hospital Stanford built a platform to enable integration of MEWS and an associated alert notification platform into the Epic EMR (Epic Systems Corporation, Verona, Wisconsin, USA) for labor and delivery and maternity units. MEWS criteria, created by the National Partnership for Maternal Safety [4], were chosen as the designated screening index for our level IV obstetric unit with 4500-5000 deliveries/year (approximately 70% high-risk). 

**Figure 1:**
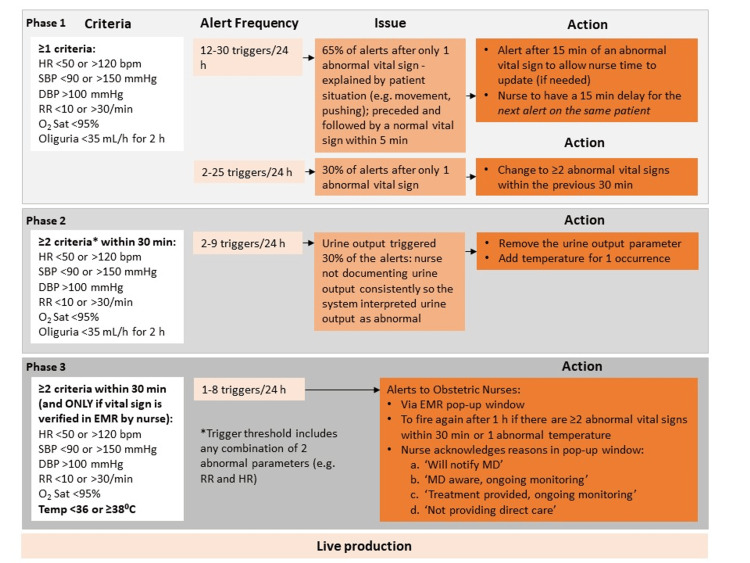
Phases of development and audit results of the maternal early warning system triggers and alert notification platform HR: heart rate; SBP: systolic blood pressure; DBP: diastolic blood pressure; RR: respiratory rate; MD: Medical doctor; EMR: electronic medical record *Mhyre et al., 2014 [4]

The acceptable alert frequency was determined to be <10 alerts/day, based on departmental consensus for tolerance, representing an alert frequency of approximately one alert/patient for this delivery volume (Figure 1). Our operational design was implemented around time periods during which nurses collect vitals at our institution; within one hour after the start of care and at a minimum every four hours for the following parameters: blood pressure (BP), heart rate (HR), respiratory rate (RR), oxygen saturation, and temperature. For patients with an epidural, BP and HR are monitored every 15 minutes, and temperature is monitored hourly after rupture of membranes. After delivery, vital signs are assessed at a minimum of every 15 minutes for two hours or until discharge from the immediate recovery period. BP, HR, and oxygen saturation data are automatically (electronically) entered in a flowsheet in the Epic EMR from the Philips Avalon (Koninklijke Philips N.V., Amsterdam, Netherlands) bedside fetal and maternal monitor. RR, temperature, and urine output are manually recorded and entered in the Epic flowsheet by the nurse. Abnormal vital signs (as per MEWS criteria) are displayed in red font, and normal vital signs are displayed in black font in the Epic flowsheet.

During the development phases, background audits of the alert notification platform were performed by CIT to determine the alert frequency and detect system issues (providers/nurses did not receive alerts during the development phases). We aimed to design an alert system to incorporate bedside monitor vitals into an automated EMR “pop-up” message that would initially alert the bedside nurse when logged into the EMR via an Epic Best Practice Advisory (Epic Systems Corporation). Following message validation in the EMR by the nurse, the alert notification provided four options: (i) Medical doctor (MD) aware, ongoing monitoring; (ii) Will notify MD; (iii) Treatment provided, ongoing monitoring; (iv) Not providing direct care. The alert system was trialed only in the intrapartum setting for laboring patients and did not include antepartum/postpartum patients. 

The original MEWS parameters are listed in Figure 1 (phase 1); however, ‘Maternal agitation, confusion, or unresponsiveness’ and ‘Patient with preeclampsia reporting a non-remitting headache or shortness of breath’ were excluded due to difficult identification in an EMR. We do not have data to report how many nurses were involved in receiving the alerts or how many patients triggered the alerts.

## Results

The impact of E-MEWS and threshold/timeframe trigger adjustments on the alert notification frequency is shown in Figure 1. Modifications and timeframe adjustments for phases 2 and 3 were implemented to reduce too frequent alert notifications that the audit considered unnecessary or inconsequential (due to artifacts resulting in inaccurate results). Specifically, the criterion in isolation resulted in too frequent alerts, some of which were due to artifacts, so ≥2 criteria were introduced in addition to a 30-minute timeframe for a subsequent alert to occur.

Analysis of data from January 1, 2019, to December 31, 2020, found 2643 alert notifications with a mean frequency of 7.2/day (range 5.6-8.5). Table 1 displays the results for each alert acknowledgement type. There was high compliance by the nurses to acknowledge alerts, with <1% of alerts not acknowledged.

**Table 1:** Electronic medical record alert acknowledgement options for nurses and frequency of alert acknowledgement types (total number of alerts = 2643) MD: Medical doctor

Alert acknowledgement type	Frequency (Percentage)
MD aware, ongoing treatment	1368 (52%)
Will notify MD	751 (28%)
Treatment provided, ongoing monitoring	425 (16%)
Not providing direct care	86 (3%)
Alert not acknowledged	13 (<1%)
Total acknowledgements	2643

## Discussion

Automated surveillance systems have shown promise in predicting maternal morbidity in the obstetric setting, and integration of MEWS into the EMR as an automated alert notification has demonstrated a reduction in paging frequency compared to paging for isolated vital sign abnormalities [7,8].

This study demonstrates the number of alerts realized, and several adjustments were necessary to ensure successful implementation. Collaboration with CIT experts in the planning and design of the system allowed for timely adjustments during scheduled audits, facilitating trigger and time delay modifications (Figure 1).

This alert system was designed as a fail-safe system when abnormal vitals are not detected by a healthcare provider. Although we show that for 68% of alerts (Table 1), nurses responded with "MD aware, ongoing treatment”, nursing and OB teams did not have to wait for an alert to initiate treatment.

In the operational design of E-MEWS, the system underwent three development phases. The first obstacle was the high frequency of alert notifications (up to 30 alerts/day) during phase 1, the majority of which were due to artifacts (patient movement or pushing, etc.). Our experience demonstrates that standard MEWS criteria cannot be inserted into EMR surveillance without criteria and time delay modifications without alert overload. Subsequent modification included a time delay of 15 minutes between detected abnormal vital signs, but this still resulted in high alert frequency. The threshold trigger of ≥1 abnormal vital signs was increased to ≥2 within the preceding 30 minutes, which also represents a previously described time-alert threshold for notification in the obstetric ward [9]. In phase 2, although the alert frequency (up to nine alerts/day) had significantly decreased, it was noted that 30% of alerts resulted from inconsistent urine output measurements, leading to system interpretation of an abnormal parameter, so urine output was removed from E-MEWS.

We acknowledge several limitations in our study design. Since our operational design was intended to measure the functionality of an automated system, outcomes associated with triggering events or impact on clinician management were not measured. We did not intend to measure triggering events in the antenatal or postpartum units by design; however, we acknowledge that a significant amount of morbidity occurs during this period and should be included in future studies. Furthermore, we did not analyze the alert frequency for each criterion, and acknowledging this information would help in future reiterations and phase development. We are cautious to automatically confer nursing compliance with a lack of alarm fatigue, as we are aware that simple acknowledgement of vital sign parameters (or other message alerts) does not always equate to reading alerts. We did not collect patient-level data, including comorbidities and conditions that led to the automated triggers. Future studies are required to evaluate whether this novel automated MEWS notification system improves patient outcomes and clinical management.

Our experience presented several challenges. The degree of high alert frequency and potential for ‘alert fatigue’ as previously described with MEWS and other surveillance alert systems, represents a potential weakness of surveillance tools [10]. Although E-MEWS led to a mean of 7.2 alerts/day, it is unclear whether modifying the system to reduce alerts will reduce the sensitivity to detect critical illness. Other vital sign screening indices have shown an overall lack of predictive value when identifying maternal critical illness [11]. Owing to this limitation, temperature (<36⁰ or ≥38⁰ Celsius) was added to E-MEWS as a recommended expert screening criterion [12].

Communication breakdown is a leading factor in maternal morbidity and mortality and sentinel event reports [13]. Of the alert notification acknowledgments, 28% were "Will notify MD". Without having the alert system, it is uncertain if a nurse would have in fact notified the Obstetric team (we did not collect data regarding conditions triggering each category). Only verified alerts contributed as potential triggers, introducing the possibility of delays or response errors [14]. Future reviews should determine the frequency of verification delays/errors, and the frequency of "will notify MD" that resulted in MD notification. Using vital signs as a preventative strategy will not change outcomes unless screening criteria are paired with interventions.

## Conclusions

This study demonstrates the logistics involved in the implementation of an alert notification platform associated with MEWS in an EMR, and potential modifications to optimize operational functionality. Integration of screening indices with automated EMR platforms represents an important step forward in the strategy to address maternal morbidity and mortality. The optimal MEWS would have high sensitivity to avoid missing cases, and high specificity to reduce alarm fatigue and, consequently, providers potentially ignoring alerts.

E-MEWS represents a valuable tool to refine MEWS. We hope future studies will examine the impact on patient outcomes and preventability of obstetric morbidity and mortality with electronic-based MEWS and alert notification platforms in antepartum, laboring, and postpartum patients, and also in different-sized hospitals and locations with varying resources and patient populations.
